# Methadone-Associated Delirium in Oncology and Palliative Care: A Clinical Perspective

**DOI:** 10.7759/cureus.110333

**Published:** 2026-06-05

**Authors:** Nandan M Shanbhag, Mehad Araki

**Affiliations:** 1 Radiation Oncology/Palliative Care, Tawam Hospital, Al Ain, ARE; 2 Nursing/Palliative Care, Tawam Hospital, Al Ain, ARE

**Keywords:** advanced cancer, cancer pain, delirium, hallucinations, methadone, neuropsychiatric toxicity, opioid-induced neurotoxicity, opioid rotation, pain management, palliative care

## Abstract

Methadone is widely used in cancer pain management, particularly when other opioids provide poor analgesia or unacceptable adverse effects. Although it is often considered less likely to cause classic opioid-induced neurotoxicity because it lacks active metabolites, available evidence suggests that methadone can still contribute to delirium and hallucinations in selected patients.

The published literature does not support a single toxic dose threshold. Reported neuropsychiatric toxicity appears to depend more on clinical context than on dose alone, especially during opioid rotation, dose escalation, or addition to existing opioid therapy. Previous opioid exposure, drug interactions, accumulation, frailty, dehydration, infection, metabolic disturbance, and central nervous system disease all complicate attribution.

At the same time, methadone is also used to improve opioid-related toxicity when patients are switched from other opioids. This creates an important clinical tension: methadone may either relieve or contribute to neuropsychiatric symptoms depending on the setting. A limited review of the literature suggests that methadone should be regarded as a plausible but not exclusive cause of delirium or hallucinations in patients with cancer. Careful monitoring is particularly important during initiation and conversion, when early neuropsychiatric symptoms may signal toxicity in a vulnerable patient.

## Editorial

Methadone is a well-established option for cancer pain when first-line opioids give poor analgesia or unacceptable adverse effects. Its lack of known active metabolites has often made it seem less likely than morphine or hydromorphone to produce classic opioid-induced neurotoxicity. This clinical advantage is real; however, it does not eliminate the possibility of methadone-associated delirium, hallucinations, or broader cognitive toxicity in advanced cancer care [1,2].

The cancer literature supports a careful middle position. Methadone can plausibly contribute to hallucinations or delirium, yet attribution is rarely simple because these syndromes usually arise in medically fragile patients with multiple competing causes. Across reviews of opioid neurotoxicity in cancer, the recurring syndromes are confusion, cognitive failure, delirium, hallucinations, sedation, myoclonus, and hyperalgesia. Isolated delusions are described less often than delirium or hallucinations and are usually folded into a broader neuropsychiatric picture rather than treated as a separate opioid-specific phenotype [3-5].

The main interpretive problem is that advanced cancer itself creates a dense background of delirium risk. Dehydration, infection, renal or hepatic dysfunction, metabolic disturbance, central nervous system disease, and concomitant psychoactive drugs all recur as alternative or additive explanations in the literature [3,6,7].

Direct evidence from the cancer and palliative care literature

The direct methadone literature is small and leans heavily on case reports, switching series, and broader opioid neurotoxicity cohorts (Table 1).

**Table 1:** Methadone-Associated Delirium and Neurotoxicity in Cancer and Palliative Care: Evidence from Case Reports and Clinical Series Summary of published evidence describing methadone as a potential contributor to delirium, hallucinations, and opioid-induced neurotoxicity in cancer and palliative care settings. The table contrasts individual case reports, where temporal association and dechallenge may support causality, with cohort and rotation studies showing that methadone-related neurotoxicity is uncommon and that methadone may also improve opioid toxicity when used as a rotation strategy. Dose, timing, and attribution should be interpreted cautiously because of co-administered opioids, opioid conversion effects, comorbid illness, and concomitant medications.

Paper	Year of publication	Number of subjects/patients	Setting	Methadone signal	Dose and timeline details	Main implication
Hoff et al. [8]	2017	One patient (case report)	Advanced cancer case report	Methadone-induced neurotoxicity is reported directly	Detailed dose and onset not available from project metadata	Strong face validity that methadone can be the offending opioid
del Rosario et al. [9]	2001	One patient (case report)	Rotation from transdermal fentanyl to oral methadone in lung cancer with bone metastases	Reversible hyperactive delirium during switch	Fentanyl 300 µg per hour plus rescue morphine before switch. Oral methadone started at 40 mg three times daily, later increased to 50 mg three times daily. Mild behavioral change by day 2, confusion and agitation by day 3, clear delirium by day 4 [9]	Shows methadone-containing regimens can plausibly trigger delirium during conversion, though carryover fentanyl and comedications complicate attribution
Ikegaki and Kizawa [10]	2020	One patient (case report)	Case report	Delirium after methadone addition	Prolonged delirium reported after a single 5 mg methadone dose added to ongoing opioid therapy [10]	Suggests that susceptible patients may develop toxicity even at very low added doses
Lim et al. [7]	2018	390 patients	Inpatient palliative care cohort of patients with advanced cancer on opioids	Methadone is listed as the primary offending opioid in a small subset	Opioid-induced neurotoxicity occurred in 15%. Among affected patients, delirium occurred in 47% and hallucinations in 40%. Methadone was the primary offending opioid in 3 of 57 cases [7]	Methadone-related neurotoxicity appears uncommon but credible within the broader opioid neurotoxicity syndrome
Parsons et al. [11]	2010	189 patients	Outpatient cancer pain initiation and rotation series	No overall rise in hallucinations or delirium after methadone start or rotation	Usual initiation was 5 mg twice daily. Median dose at first follow-up was 10 mg in initiation patients and 15 mg in rotation patients. First follow-up was around day 13 [11]	Methadone often improves rather than worsens opioid toxicity, so causation must be argued from case-specific timing and dechallenge
Mercadante et al. [12]	2005	31 patients	Rapid switch between fentanyl and methadone	Confusion and drowsiness often improved after the switch	The initial fentanyl-to-methadone ratio was 1 to 20. Mean time to stabilization after fentanyl to methadone switch was 4.3 days [12]	Supports methadone as a therapeutic rotation option, while also showing that the early post-switch period is clinically unstable

Dose and timeline patterns

No paper in this set establishes a reliable toxic methadone threshold for hallucinations or delirium in adult patients with cancer. The evidence instead shows a wide spread of potentially risky circumstances.

At one end, the most striking low-dose signal comes from the report of prolonged delirium after a single 5 mg dose added to an existing oxycodone regimen [10]. At the other end, the clearest rotation-associated signal comes from the fentanyl to methadone case, in which delirium emerged after the initiation at 120 mg per day during a high-dose opioid switch and became definite by day 4 [9]. These reports argue against any simple rule that toxicity appears only above a fixed daily dose.

The broader methadone literature helps explain why. Methadone conversion ratios are variable and become harder to predict in opioid-tolerant patients. In a prospective cancer pain study of switching from morphine to oral methadone, the morphine to methadone ratio ranged from 2.5 to 1 up to 14.3 to 1, with a median of 7.75 to 1, and a median of three days was needed to reach equianalgesia [13]. Reviews of methadone in cancer pain likewise stress its long and unpredictable half-life and the risk of accumulation during initiation or rotation [1,14]. The practical implication is that dose cannot be interpreted apart from previous opioid burden, conversion method, rescue dosing, and the first few days after the switch.

Timeline patterns are more consistent than dose patterns. The most convincing reports place the onset in the first hours to several days after methadone initiation, addition, or rotation [9,10]. Larger clinical series are less granular; however, they still point to an early vulnerable window. In the outpatient series, the first formal follow-up occurred about 13 days after methadone start or rotation, and the authors specifically warned about delayed sedation and drug interaction-driven toxicity even when the overall cohort did not show rising delirium rates [11]. In the rapid switch study, dose stabilization after fentanyl to methadone conversion took a mean of 4.3 days, again suggesting that the first several days after conversion are the key period for neurotoxicity surveillance [12].

Why is attribution hard?

The best counterweight to a simple methadone causation claim is that the same literature repeatedly shows methadone being used to relieve neurotoxicity from other opioids. In the outpatient cancer series, hallucinations and delirium did not increase after methadone initiation or rotation, and many patients improved clinically on methadone [11]. In the prospective morphine to methadone study, switching was effective in most patients and often improved adverse effects, while pain control was regained over about 3.5 days [15]. In the rapid fentanyl switch study, confusion and drowsiness generally improved rather than worsened after conversion [12].

This dual role matters in a case discussion. Methadone is neither uniformly protective nor uniformly toxic. It is better understood as an opioid whose benefits and risks are highly context dependent. The strongest argument for causation, therefore, comes from the clinical pattern rather than from the drug name alone.

Several recurring confounders deserve explicit mention. Reviews of opioid neurotoxicity in cancer identify renal impairment, high opioid dose, prolonged opioid exposure, dehydration, baseline cognitive vulnerability, and co-administration of psychoactive drugs as common correlates of central nervous system irritability [3]. Earlier series of narcotic-associated mental status impairment in metastatic cancer also emphasize fever, infection, other central nervous system depressants, and dose conversion problems as causes of mental status change that may mimic pure opioid toxicity [6]. In the fentanyl to methadone delirium case, amitriptyline and dexamethasone were present, and the authors ultimately favored additive opioid overdose from residual fentanyl plus newly started methadone rather than methadone alone [9]. 

Discussion points that support methadone as a plausible contributor

A methadone-related explanation is the strongest when symptoms begin soon after initiation, dose increase, or opioid rotation [9,10]. A convincing dechallenge pattern is improvement after methadone reduction, discontinuation, or opioid change, even if other delirium treatments are also given [7]. Previous high opioid exposure matters because methadone conversion is nonlinear, and residual opioid effect can overlap with newly introduced methadone [9,13]. Low-dose methadone does not exclude causation in a vulnerable patient, particularly when it is added to an existing opioid regimen rather than used in isolation [10]. A claim that methadone was involved is more defensible than a claim that methadone was the sole cause when dehydration, infection, metabolic derangement, brain disease, or psychoactive comedications are present [3,6,7].

Conclusions

The published cancer and palliative care literature supports methadone as a plausible cause or contributor to hallucinations and delirium, but not as a drug with a clear toxic dose threshold. The most useful evidence comes from temporal association, conversion context, and reversibility, not from any single dose cutoff. At the same time, the same literature shows that methadone often improves opioid-related neurotoxicity when patients are switched from other opioids. A more extensive systematic review of literature is needed to explore this less-known possible adverse effect.
